# Prevention and control strategies for the diagnosis and treatment of cancer patients during the COVID-19 pandemic

**DOI:** 10.1038/s41416-020-0854-2

**Published:** 2020-04-20

**Authors:** Juan Tan, Chao Yang

**Affiliations:** 1 Outpatient Department, Children’s Hospital of Chongqing Medical University, Lijia Campus, Chongqing, 401122 China; 2 Department of Surgical Oncology, National Clinical Research Center for Child Health and Disorders, Ministry of Education Key Laboratory of Child Development and Disorders, China International Science and Technology Cooperation Base of Child Development and Critical Disorders, Children’s Hospital of Chongqing Medical University, Chongqing, 400014 China

**Keywords:** Cancer, Health policy

## Abstract

COVID-19 has had a major impact worldwide due to its high infectiousness. Patients with cancer are more susceptible to infection and more likely to have severe events than other patients. This paper proposes management strategies for cancer patients that are beneficial for pandemic control and reduce the impact of the pandemic on cancer patients.

## Main

Since the outbreak of a novel coronavirus (SARS–CoV-2) infection in Hubei in December 2019, SARS–CoV-2 has spread throughout China and worldwide.^1^ The population is generally susceptible, and elderly individuals and those with underlying diseases are more likely to present with severe disease.^1,2^ The World Health Organisation (WHO) announced that the novel coronavirus disease (COVID-19) caused by SARS–CoV-2 is a public health emergency of international concern and raised the global risk level to the highest level. By 15 April, 2020, 1,878,489 confirmed cases had been reported in more than 200 countries, resulting in 119,044 deaths. The worldwide epidemic of SARS–CoV-2 may be a once-in-a-century pandemic,^3^ so how to deal with the epidemic and protect public health is the first priority. Patients with cancer are more susceptible to infection and are more likely to have severe events due to the immunosuppressive state caused by cancer itself and anticancer treatments, such as chemotherapy, radiotherapy or surgery.^4^ Indeed, Wenhua Liang and his colleagues found that the incidence of COVID-19 patients with a history of cancer was higher than the incidence of cancer in the overall Chinese population, and that patients with cancer were observed to have a higher risk of severe events than other patients.^5^ Therefore, it is a matter of great concern for every patient and medical staff member to co-ordinate epidemic control and cancer treatment during the epidemic period. Considering the characteristics of SARS–CoV-2 transmission and the requirements for prevention and control of COVID-19 by relevant departments, this paper proposes strategies for cancer diagnosis and treatment during the pandemic that can provide information for SARS–CoV-2 prevention and control, reduce the impact of the pandemic on cancer diagnosis and treatment and ensure the continuity of treatment.

## Diagnosis and treatment principle of cancer based on the prevalence of COVID-19

### Surgery consideration

We recommend stratifying patients who need surgery as needing an emergency, confined or selective operation based on the situation. Regardless of whether tumours are benign or malignant, if there is rapid progress, severe organ function impairment or a life-threatening situation, such as rupture and haemorrhage of the tumour or severe compression caused by a large tumour, an emergency operation should be considered. A confined operation should be comprehensively evaluated and reasonably arranged according to the local epidemic and hospital situation. Tumour resection after neoadjuvant chemotherapy should be completed in as reasonable an interval as possible if all conditions permit. By contrast, a radical operation should be postponed until the end of the epidemic.

In patients with a benign tumour and stable clinical presentation without severe organ or airway compression, a watch-and-see approach is an option to avoid the peak of the epidemic. Patients with an unclear benign/malignant tumour should be diagnosed by biopsy in advance, and it should be noted that the biopsy operation is considered a confined operation when malignancy is highly suspected. We suggest initiating neoadjuvant chemotherapy after biopsy, clinical stage and risk classification. Upfront high-risk radical surgery during the epidemic should be avoided.

During the epidemic period, there may be a shortage of blood supplies, so blood preparation is required before an operation. Due to the different severities of the epidemic, management and isolation policies, it is recommended that patients who need surgery stay in the hospital near their own low-risk area for treatment.

### Chemotherapy and radiotherapy arrangements

Chemotherapy and radiotherapy are important components of comprehensive treatment for cancer. The prognosis of patients is closely related to how to ensure the standardisation and continuity of chemotherapy and radiotherapy during the pandemic. After chemotherapy, the majority of patients will present with myelosuppression of varying degrees and durations, which will increase the risk of infection. Severe thrombocytopenia may lead to fatal bleeding, and the risk of the patient is even higher due to the blood shortage during the pandemic. Chemotherapy in local hospitals is preferred to reduce population migration. High-dose chemotherapy and other regimens with severe myelotoxicity should be avoided. Dose-reduction chemotherapy is an option if necessary. In some cases, oral chemotherapy is a feasible choice, especially for palliative treatment. Radiotherapy might cause myelosuppression and increase the risk of infection due to increased exposure. It is recommended that the radiotherapy be postponed if not necessary during the pandemic.

### Management of the oncology clinic and ward during the pandemic

*Outpatient department*: We recommended establishing a “triple pre-examination” system, which consists of “entrance of the hospital”, “general area” and “cancer clinic area”, for preparative checking and dividing. Patients with no history of epidemiological exposure and no symptoms such as fever can be allowed to enter the oncology clinic; otherwise, patients should be transferred to the isolation clinic, where oncologists can go for consultation. Establishing and following a uniform hospital admission process for cancer patients is recommended. A complete blood count and chest radiography should be obtained at the clinic.

*Inpatient department*: All infection control regulations and control measures for COVID-19 must be strictly implemented.^6,7^ The number of medical staff in the ward should be limited. According to the risk of exposure of different positions, stratified protection measures should be taken to reduce the risk of nosocomial infection. Based on Chinese hospital experiences, we recommend establishing three areas (a clean area, a potential contaminated area and a contaminated area), two channels (a medical staff channel and a patient channel) and an isolation room in the oncology department. Adequate disinfection should meet the corresponding guideline requirements.

### Adjustment of clinical trials

All trials ready for review should be submitted online. Initiation of newly approved programmes should be postponed. Recruitment for initiated studies should be suspended. For the subjects who needed to come to the hospital for follow-up recently, in case of serious adverse events, the visiting team needs to know the epidemiological history for the past 14 days, including fever, cough and other conditions, and provide corresponding medical advice and help. It is recommended that subjects go to the local research centre for regular follow-up. If subjects going to the study centre for medication cannot be obtained, the team should mail the medication to the subject in accordance with the drug retention standards. During the pandemic, if a breach occurs for any reason, the investigator should communicate fully with the sponsor, record it truthfully and report the situation to the ethics committee.

## Data Availability

Not applicable.
